# Patient and course of care factors impacting time to response and remission in psychotherapy for depression

**DOI:** 10.3389/fpsyg.2026.1816893

**Published:** 2026-08-11

**Authors:** Nicholas R. Forand, Jasmine Nettiksimmons, Carol Hardy, Margaret Anton, Brandn Green

**Affiliations:** 1 Two Chairs, San Francisco, CA, United States; 2 Department of Psychiatry, Donald and Barbara Zucker School of Medicine at Hofstra/Northwell, Hempstead, NY, United States; 3 JG Research & Evaluation, Bozeman, MT, United States

**Keywords:** case complexity, depression, measurement-based care, predictive modeling, psychotherapy, remission, response, treatment duration

## Abstract

**Introduction:**

Psychotherapy effectively treats depression, but variation exists in response timing and treatment trajectories.

**Methods:**

Using data from 7,161 adults receiving psychotherapy for depression (baseline PHQ-9 ≥10), we examined how baseline patient characteristics predict time to first response (≥50% symptom reduction) and first remission (PHQ-9 < 5), and whether patient features predict later outcomes for non-responders after 24 psychotherapy sessions. Baseline predictors were identified using elastic net; time to outcomes used Poisson regression.

**Results:**

Overall, 67.9% achieved first response and 45.6% achieved first remission at median times of 6 and 8 sessions, (respectively); most responses occurred by session 12 (81.9%). Features predicting faster response included adjustment disorder diagnosis and good health; slower response related to comorbid anxiety, chronic pain, and diagnostic complexity. Among non-responders by session 24 (*n* = 778: first response, *n* = 1,477 first remission), only early symptom trajectories—not baseline characteristics—predicted later response.

**Discussion:**

Findings support using complexity assessments for initial treatment planning and ongoing symptom monitoring for mid-treatment adjustments.

## 1 Introduction

Major depressive disorder (MDD) is estimated to affect 19.8 million adults in the United States alone (Greenberg et al., 2023), and the presence of depression is associated with increased burden across home, community, work and school settings (World Health Organization, 2023). Psychotherapy is established as an effective treatment for depression (Cuijpers, 2017; Cuijpers et al., 2023), with benefits observed across diverse populations and treatment modalities (Cuijpers et al., 2014). The literature also demonstrates meaningful healthcare cost-savings for patients receiving successful clinical treatment (Simon et al., 2006; Popkov et al., 2025). While psychotherapy demonstrates strong average effects, substantial heterogeneity exists in individual response patterns, leaving clinicians uncertain about expected treatment response trajectories for specific patients (Owen et al., 2015; Saunders et al., 2019). This heterogeneity leaves providers and health systems with a lack of clarity about treatment plans, specifically, when to adjust the treatment plan due to lack of response, or when to continue with the original treatment plan.

The dose-response and good-enough level (GEL) models offer complementary perspectives on the effective dose of psychotherapy. Dose-response research examines the association between therapy sessions (“dose”) and symptom improvement. Typical dose-response effects for depression treatment show rapid early change and diminishing marginal gains with each additional session (Robinson et al., 2020; Lee et al., 2021). However, this model assumes relative uniformity in session-by-session gains across patients. GEL models address this limitation by proposing that patients improve to individually meaningful thresholds before plateauing, thereby accommodating the heterogeneity in treatment trajectories driven by baseline severity, socioeconomic factors, and other individual characteristics (Stulz and Lutz, 2007; Lee et al., 2021).

In a review of the dose-response and GEL literature, Robinson et al. (2020) found that the optimal number of sessions identified across studies ranged widely between 4 to 26 sessions across both approaches for accounting for an effective dose of psychotherapy. Factors influencing this number included the frame of therapy (e.g., time-limited vs. open-ended), clinical settings, and populations under study. While these session ranges are helpful for understanding when the average patient might expect to receive benefit, if patient individual response to care is heterogenous (consistent with the GEL model), the existing literature provides minimal guidance about which patients are likely to benefit and by when in their course of care (Stulz et al., 2013; Robinson et al., 2020; Lee et al., 2021; Klein et al., 2024).

A common approach to the lack of clinical response is for the provider to continue therapy until a positive outcome is achieved or the patient terminates care (Stewart and Chambless, 2008; American Psychological Association, 2019). While many patients may appreciate the prolonged therapeutic relationship, this approach has tradeoffs: it is costly without guaranteeing a positive outcome and consumes a valuable slot on a provider's schedule. Indeed some authors have found that the likelihood of a positive response is negatively correlated with the number of sessions attended, suggesting that prolonged treatment may not improve odds of a good outcome (Stiles et al., 2015). Clinicians will benefit from efforts to expand evidence-based guidance about who is likely to respond later in care, as this understanding can inform decisions about when the best course of action is to continue treatment, to modify the treatment approach, or to refer patients to alternative levels of care.

Patient characteristics associated with treatment prognosis may help improve our understanding of how long patients need to be in care. Several patient characteristics have been found to predict depression treatment outcomes. Sociodemographic factors including income, employment status, relationship status, and health insurance type are associated with treatment response trajectories, with lower socioeconomic status predicting slower improvement (Perlman et al., 2019; Mills et al., 2022). Clinical severity indicators, including suicidal ideation, severe depression, and comorbidity, predict worse outcomes (de Beurs et al., 2020; Buckman et al., 2021; Perlman et al., 2019; Riera-Serra et al., 2024).

Moving beyond univariate associations, models assessing “case complexity” or the cumulative effect of patient characteristics across clinical, demographic, and characterological, and dispositional domains may provide a stronger prediction of clinical outcome. In Delgadillo et al. (2017), case complexity factors are hypothesized to affect treatment outcomes by overwhelming patient coping resources or by disrupting engagement in treatment. These authors argue that case complexity can be conceptualized as the cumulative detrimental effect of risk factors on treatment outcomes (potentially offset by co-occurring patient resiliency or protective factors). These authors demonstrated with a statistical model that quantified complexity predicted outcomes and informed treatment intensity recommendations, with complex cases benefiting more from a higher-intensity care model. The utility of case complexity in making initial clinical decisions about the intensity of care was later supported in a cluster randomized trial (Delgadillo et al., 2022).

Both univariate and cumulative prognostic factors have been studied as predictors of end-of-treatment outcomes in controlled and naturalistic settings, but their associations with time-to-response are less well characterized (Anderson and Lambert, 2001; Hansen and Lambert, 2003). Further, little research addresses conditional prediction—identifying which patients who remain symptomatic after substantial treatment are likely to benefit from continued care.

Another approach to understanding the duration of treatment required for patients to achieve a clinical response has been to examine the trajectory of symptom change early in care. Several studies have found that early rapid response predicts better longer-term outcomes (Lutz et al., 2009; Lewis et al., 2012; Li et al., 2023). Other evidence suggests that the variability of symptoms over time, particularly pre-treatment, is also associated with a more favorable outcome (Bosley et al., 2019). Together, this literature suggests that individuals whose symptoms are more dynamic or pliable may be more likely to respond to treatment. Similar to the complexity literature, however, most trajectory research has focused on early in care trajectories and their prediction of end of care outcomes. Little research has focused on whether treatment trajectory information can be used to determine the likely benefit for patients who have already received an effective “average” dose of care.

In summary, patients receiving psychotherapy for depression demonstrate heterogeneity in both overall response and time to response. Minimal guidance exists for clinicians to help determine who may receive benefit from continued care after the “optimal duration” of care has passed. Case complexity has been used as means to predict overall response to depression care and may offer helpful guidance for clinicians and other stakeholders in predicting time to response. This information may be supplemented by patterns of early response, which may help update predictions about the potential benefit of continued psychotherapy. However, there remain key gaps in the research literature about the relationship between time to response and case complexity.

Given the substantial disability, morbidity, and cost associated with depression, information that can guide expectations for treatment duration and outcomes can be useful for clinicians and individuals receiving care. Such information may be leveraged to improve clinical decision-making and allocation of treatment resources. The current study is intended to address these gaps by evaluating time to response and remission in a large naturalistic sample of patients receiving psychotherapy for depression with up to 48 sessions of care. The study examined the influence of patient baseline characteristics on time to outcome, as well as predictors of later response among patients who have not achieved outcomes by session 24.

## 2 Material and methods

### 2.1 Setting

This study was conducted using data from a technology-enabled behavioral health service provider. This service provides teletherapy to adults aged 18 and older with anxiety, depression, and related conditions. Providers practice a range of therapeutic modalities including CBT, ACT, EFT, IFS, psychodynamic psychotherapy, EMDR and IPT. The use of modality is not prescribed and providers use their clinical judgment and expertise to select and deliver the appropriate intervention. Care is supported by a technology platform and comprehensive measurement-based care (MBC) system that includes session-by-session automated assessments, clinical decision support, and clinician training. The setting and system have been described in detail in previous publications (Forand et al., 2025a,b). All study procedures were deemed exempt by the Sterling Institutional Review Board, Atlanta, Georgia, United States.

### 2.2 Data collection

Patient data were collected as part of standard clinical practice via four sources: a standard onboarding questionnaire, an optional “patient profile” where individuals could complete more information related to their characteristics and care, data entered into the electronic medical record by providers, and weekly measurement-based care surveys consisting of the PHQ-9 (Kroenke et al., 2001) and GAD-7 (Spitzer et al., 2006). The MBC surveys also include a quality of life measure and a measure of therapeutic alliance; however, these are not used in the current study. Measures are sent prior to every therapy session, and measure completion rates in this sample were 95%.

### 2.3 Patient inclusion criteria

Patients who began treatment from October 1, 2023 to December 31, 2024 and whose baseline PHQ-9 score was ≥ 10 were eligible for inclusion (*N* = 7,618). The following exclusion criteria were imposed: (1) the patient must have a PHQ-9 evaluated at either the first session or at intake, excludes *n* = 22 (0.3%); (2) the patient must have at least one follow-up PHQ-9 score, excludes *n* = 348 (5%); (3) the patient must not be missing diagnostic information derived from claims, excludes *n* = 112 (1%). After these exclusions, the final analytic sample size was *n* = 7,161 (94%) of the original inclusion set (note: some patients meet more than one exclusion criterion). Patients were followed up to a maximum of 48 attended sessions, capped by 1 year of follow-up time from their first session. Data were captured on January 8, 2026.

### 2.4 Outcomes

#### 2.4.1 Measures

Patients were assessed with the Patient Health Questionnaire-9 (PHQ-9) and Generalized Anxiety Disorder-7 (GAD-7) at each session (95% completion rate). The PHQ-9 is a 9-item self-report of depressive symptoms. Items are rated on a scale ranging from 0 (not at all) to 3 (nearly every day). Total scores range from 0 to 27. The presence of suicidal ideation was defined as scoring > 0 on item 9 of the PHQ-9 at baseline. The GAD-7 is a 7-item self-report measure of anxiety. Items are rated on a scale ranging from 0 (not at all) to 3 (nearly every day). Total scores range from 0 to 21.

#### 2.4.2 Clinical outcomes

The primary outcomes of interest were presence of and time to first depression response, defined as a 50% or greater improvement in symptoms as measured by the PHQ-9. Secondary outcomes of interest were presence of and time to first depression remission, defined as PHQ-9 score < 5. Maintenance of outcomes was evaluated at the first available session at least four sessions after the initial outcome.

### 2.5 Baseline patient features

#### 2.5.1 Race and ethnicity

A patient race/ethnicity variable was defined as follows. Patients who endorsed Hispanic/Latino, regardless of selected race, were categorized as Hispanic/Latino. Patients who endorsed more than one race were classified as multiracial. Patients with missing race/ethnicity information were classified as “unknown.” Patients who solely endorsed Asian Pacific Islander or American Indian/Alaska Native were grouped together as “other” and combined with “unknown” in models due to low sample size.

#### 2.5.2 Diagnoses

Patient diagnoses were assessed using International Classification of Diseases, 10th revision (ICD-10) codes from billing claim records. To ensure clinicians had adequate time to reliably determine the diagnosis, claims were observed for 30 days after the initial session. For parsimony, diagnoses were categorized into depressive disorders, adjustment disorders, anxiety disorders, trauma or stress-related disorders, neurodevelopmental disorders, bipolar disorders, or other disorders. To examine the effect of diagnostic complexity on outcomes, a mutually exclusive diagnosis category variable was created as follows: anxiety only, depression only, depression and anxiety only, adjustment disorder only, bipolar disorder, trauma and stress disorders, neurodevelopmental disorders, and other disorders. Patients in the anxiety, depression, or anxiety and depression categories may include patients who had a preliminary adjustment diagnosis. Patients in the bipolar, trauma, and neurodevelopmental groups may also have other diagnoses; the category was selected in the priority order shown above. Patients whose claim diagnoses do not meet any of these criteria and either have solely “other” diagnoses or “other” diagnoses in addition to depression/anxiety are designated “other.” The number of diagnosis domains was calculated as an additional feature of complexity and excludes adjustment disorder in the presence of other diagnoses.

### 2.6 Analytical methods

We examined associations between baseline patient characteristics and the incident rates of initial depression response and remission, estimated as rates per unit of clinical contact across session intervals. A breakdown of baseline patient features is reported in Table 1, which also includes frequency (95% CI) of remission and response in those groups, *p*-values derived from Wald tests evaluating the association between a patient feature and response/remission, and median time to outcome in individuals who achieved outcomes.

**Table 1:** Description of study population with regard to baseline features, attainment of outcomes at any point during the study duration, median session at outcome attainment, and strength of univariate association with outcomes.

Feature	Level	Frequency *N* (%)	Response: % (95%CI)	Response: Session median (IQR)	Response: *p*-value	Remission: % (95%CI)	Remission: Session median (IQR)	Remission: *p*-value
Age	18–29 years	2,288 (32.0%)	68.0% (66.0%, 69.9%)	6 (3, 10)	0.38	45.6% (43.5%, 47.7%)	6 (3, 10)	0.21
	30–49 years^*^	3,386 (47.3%)	68.4% (66.8%, 70.0%)	6 (3, 10)		45.9% (44.2%, 47.6%)	6 (3, 10)	
	50–69 years	1,274 (17.8%)	65.9% (63.2%, 68.4%)	5 (3, 10)		44.0% (41.3%, 46.8%)	5 (3, 10)	
	70+ years	213 (3.0%)	69.5% (62.8%, 75.5%)	5.5 (4, 10)		51.6% (44.7%, 58.5%)	5.5 (4, 10)	
Gender	Female^*^	4,291 (59.9%)	68.2% (66.8%, 69.6%)	6 (3, 10)	0.04	45.8% (44.3%, 47.3%)	6 (3, 10)	0.23
	Male	1,621 (22.6%)	69.5% (67.1%, 71.7%)	6 (3, 10)		46.9% (44.4%, 49.3%)	6 (3, 10)	
	Trans/non-binary/other	418 (5.8%)	64.4% (59.5%, 68.9%)	6 (4, 11)		41.6% (36.9%, 46.5%)	6 (4, 11)	
	Unknown	831 (11.6%)	64.6% (61.2%, 67.9%)	5 (3, 9)		44.4% (41.0%, 47.9%)	5 (3, 9)	
Race/ethnicity	Asian	653 (9.1%)	71.4% (67.7%, 74.8%)	6 (3, 10)	0.07	48.1% (44.2%, 52.0%)	6 (3, 10)	0.55
	Black	463 (6.5%)	68.9% (64.4%, 73.0%)	4 (3, 8)		46.0% (41.4%, 50.7%)	4 (3, 8)	
	Hispanic	1,438 (20.1%)	68.6% (66.1%, 70.9%)	6 (3, 10)		46.5% (43.9%, 49.1%)	6 (3, 10)	
	Multiracial	475 (6.6%)	68.2% (63.8%, 72.3%)	6 (4, 10)		46.9% (42.4%, 51.5%)	6 (4, 10)	
	White^*^	2,926 (40.9%)	67.8% (66.1%, 69.5%)	6 (3, 11)		45.1% (43.3%, 46.9%)	6 (3, 11)	
	Other	66 (0.9%)	72.7% (60.2%, 82.6%)	6 (4, 10.25)		50.0% (38.3%, 61.7%)	6 (4, 10.25)	
	Unknown race/ethnicity	1,140 (15.9%)	64.2% (61.3%, 67.0%)	6 (3, 9)		43.7% (40.8%, 46.6%)	6 (3, 9)	
Profile completion	Yes^*^	6,375 (89.0%)	68.2% (67.1%, 69.4%)	6 (3, 10)	0.06	45.7% (44.4%, 46.9%)	6 (3, 10)	0.90
	No	786 (11.0%)	64.9% (61.4%, 68.2%)	5 (3, 9)		45.4% (41.9%, 49.0%)	5 (3, 9)	
Insurance	HMO^*^	5,339 (74.6%)	69.0% (67.7%, 70.2%)	6 (3, 10)	0.01	46.6% (45.3%, 47.9%)	6 (3, 10)	0.001
	Commercial/self-pay	366 (5.1%)	65.0% (59.9%, 69.9%)	5 (3, 8)		44.3% (39.1%, 49.5%)	5 (3, 8)	
	Medicaid	1,017 (14.2%)	63.7% (60.7%, 66.7%)	5 (3, 11)		40.1% (37.1%, 43.2%)	5 (3, 11)	
	Medicare	439 (6.1%)	66.1% (61.4%, 70.4%)	5 (3, 10)		47.8% (43.1%, 52.6%)	5 (3, 10)	
Relationship status	Partnered/married^*^	3,431 (47.9%)	68.9% (67.3%, 70.4%)	6 (3, 10)	0.001	46.7% (45.0%, 48.4%)	6 (3, 10)	0.004
	Separated/divorced/widowed	549 (7.7%)	72.3% (68.3%, 76.0%)	6 (3, 11)		50.6% (46.4%, 54.9%)	6 (3, 11)	
	Single	1,943 (27.1%)	67.4% (65.2%, 69.4%)	6 (3, 11)		42.9% (40.7%, 45.1%)	6 (3, 11)	
	Unknown/other	1,238 (17.3%)	63.8% (61.1%, 66.5%)	5 (3, 10)		44.8% (42.0%, 47.7%)	5 (3, 10)	
Employment status	Full-time^*^	3,620 (50.6%)	70.5% (68.9%, 71.9%)	6 (3, 10)	< 0.001	47.4% (45.7%, 49.0%)	6 (3, 10)	0.003
	Caregiver	93 (1.3%)	65.6% (54.9%, 74.9%)	5 (3, 9)		40.9% (30.9%, 51.6%)	5 (3, 9)	
	Disabled	174 (2.4%)	58.6% (50.9%, 65.9%)	6 (3, 12)		35.6% (28.6%, 43.3%)	6 (3, 12)	
	Part-time	678 (9.5%)	65.2% (61.5%, 68.8%)	6 (3, 10)		44.0% (40.2%, 47.8%)	6 (3, 10)	
	Retired	336 (4.7%)	67.6% (62.2%, 72.5%)	5 (3, 9)		48.8% (43.4%, 54.3%)	5 (3, 9)	
	Student	345 (4.8%)	64.9% (59.6%, 69.9%)	6 (4, 11)		47.5% (42.2%, 52.9%)	6 (4, 11)	
	Unemployed	620 (8.7%)	65.8% (61.9%, 69.5%)	6 (3.75, 11)		41.1% (37.2%, 45.1%)	6 (3.75, 11)	
	Unknown	1,295 (18.1%)	65.2% (62.5%, 67.8%)	6 (3, 10)		44.2% (41.4%, 46.9%)	6 (3, 10)	
Health status	Very Good/excellent	1,423 (19.9%)	75.8% (73.5%, 78.0%)	5 (3, 9)	< 0.001	55.2% (52.6%, 57.8%)	5 (3, 9)	< 0.001
	Good^*^	2,764 (38.6%)	68.3% (66.6%, 70.1%)	6 (3, 10)		46.6% (44.7%, 48.4%)	6 (3, 10)	
	Fair/poor	2,108 (29.4%)	63.2% (61.1%, 65.2%)	6 (4, 12)		37.9% (35.8%, 40.0%)	6 (4, 12)	
	Unknown	866 (12.1%)	64.5% (61.2%, 67.7%)	5 (3, 9)		45.7% (42.4%, 49.1%)	5 (3, 9)	
Family history of mental illness	No^*^	2,180 (30.4%)	71.0% (69.0%, 72.9%)	5 (3, 10)	< 0.001	49.7% (47.6%, 51.8%)	5 (3, 10)	< 0.001
	Yes	4,032 (56.3%)	67.1% (65.6%, 68.5%)	6 (3, 10)		43.7% (42.2%, 45.3%)	6 (3, 10)	
	Unknown	949 (13.3%)	64.0% (60.8%, 67.0%)	5 (3, 9)		44.4% (41.2%, 47.6%)	5 (3, 9)	
Baseline PHQ	Moderate (10–14)^*^	4,063 (56.7%)	68.6% (67.1%, 70.0%)	5 (3, 10)	0.11	53.0% (51.4%, 54.5%)	5 (3, 10)	< 0.001
	Moderately severe (15–19)	2,171 (30.3%)	67.7% (65.7%, 69.7%)	6 (3, 10)		39.5% (37.4%, 41.6%)	6 (3, 10)	
	Severe (20–27)	927 (12.9%)	64.9% (61.8%, 68.0%)	6 (3, 10.75)		27.9% (25.1%, 31.0%)	6 (3, 10.75)	
Baseline GAD-7	Minimal (0–4)^*^	268 (3.7%)	72.8% (66.9%, 77.9%)	4 (3, 8)	< 0.001	56.0% (49.8%, 62.0%)	4 (3, 8)	< 0.001
	Mild (5–9)	1,878 (26.2%)	71.1% (69.0%, 73.1%)	5 (3, 9)		54.0% (51.8%, 56.3%)	5 (3, 9)	
	Moderate (10–14)	2,776 (38.8%)	68.1% (66.3%, 69.8%)	6 (3, 11)		45.8% (43.9%, 47.7%)	6 (3, 11)	
	Severe (15–21)	2,239 (31.3%)	64.3% (62.2%, 66.3%)	6 (3.5, 11)		37.2% (35.2%, 39.2%)	6 (3.5, 11)	
Baseline PHQ-9	No suicidal ideation^*^	5,226 (73.0%)	67.5% (66.2%, 68.7%)	6 (3, 10)	0.23	46.3% (44.9%, 47.6%)	6 (3, 10)	0.08
	Any suicidal ideation	1,935 (27.0%)	68.9% (66.8%, 71.0%)	5 (3, 10)		43.9% (41.7%, 46.2%)	5 (3, 10)	
Diagnosis group	Adjustment disorder only	425 (5.9%)	72.7% (68.2%, 76.8%)	5 (3, 8)	< 0.001	55.1% (50.2%, 59.8%)	5 (3, 8)	< 0.001
	Anxiety disorder only	758 (10.6%)	70.3% (66.9%, 73.5%)	5 (3, 9)		52.4% (48.8%, 56.0%)	5 (3, 9)	
	Depression and anxiety	2,321 (32.4%)	69.1% (67.2%, 71.0%)	6 (3, 11)		44.7% (42.6%, 46.7%)	6 (3, 11)	
	Depression only^*^	931 (13.0%)	70.5% (67.4%, 73.4%)	6 (3, 11)		47.7% (44.4%, 51.0%)	6 (3, 11)	
	Bipolar	269 (3.8%)	61.3% (55.2%, 67.1%)	6 (3, 12)		39.0% (33.2%, 45.2%)	6 (3, 12)	
	Neurodevelopmental	473 (6.6%)	63.2% (58.7%, 67.5%)	6 (4, 10.5)		43.8% (39.3%, 48.4%)	6 (4, 10.5)	
	Trauma and stressor	1,258 (17.6%)	64.7% (62.0%, 67.3%)	5.5 (3, 11)		41.5% (38.8%, 44.3%)	5.5 (3, 11)	
	Other	726 (10.1%)	66.0% (62.4%, 69.4%)	5 (3, 9)		44.4% (40.7%, 48.1%)	5 (3, 9)	
Number of diagnoses	1^*^	2,672 (37.3%)	70.2% (68.4%, 71.9%)	5 (3, 10)	< 0.001	49.7% (47.8%, 51.6%)	5 (3, 10)	< 0.001
	2	3,582 (50.0%)	67.4% (65.9%, 68.9%)	6 (3, 10)		44.7% (43.0%, 46.3%)	6 (3, 10)	
	3	806 (11.3%)	62.9% (59.5%, 66.2%)	6 (3, 11)		37.8% (34.5%, 41.3%)	6 (3, 11)	
	4	96 (1.3%)	60.4% (49.9%, 70.1%)	5.5 (4, 11)		33.3% (24.2%, 43.8%)	5.5 (4, 11)	
	5	5 (0.1%)	60.0% (17.0%, 92.7%)	6 (4.5, 7)		60.0% (17.0%, 92.7%)	6 (4.5, 7)	
Diabetes	No^*^	6,807 (95.1%)	68.0% (66.9%, 69.1%)	6 (3, 10)	0.24	46.0% (44.8%, 47.2%)	6 (3, 10)	0.01
	Yes	354 (4.9%)	65.0% (59.7%, 69.9%)	6 (4, 11)		39.0% (33.9%, 44.3%)	6 (4, 11)	
Chronic pain	No^*^	5,949 (83.1%)	69.5% (68.3%, 70.6%)	5 (3, 10)	< 0.001	47.5% (46.2%, 48.8%)	5 (3, 10)	< 0.001
	Yes	1,212 (16.9%)	59.9% (57.1%, 62.7%)	7 (4, 12)		36.4% (33.7%, 39.2%)	7 (4, 12)	
Heart disease	No^*^	7,061 (98.6%)	68.1% (67.0%, 69.2%)	6 (3, 10)	0.001	45.8% (44.7%, 47.0%)	6 (3, 10)	0.002
	Yes	100 (1.4%)	51.0% (40.9%, 61.1%)	7 (4, 11.5)		31.0% (22.3%, 41.1%)	7 (4, 11.5)	
Cancer	No	6,963 (97.2%)	67.9% (66.8%, 69.0%)	6 (3, 10)	0.61	45.7% (44.5%, 46.8%)	6 (3, 10)	0.84
	Yes	198 (2.8%)	66.2% (59.1%, 72.6%)	5 (3, 9)		44.9% (37.9%, 52.2%)	5 (3, 9)	
Respiratory problems	No^*^	6,606 (92.2%)	68.1% (67.0%, 69.2%)	6 (3, 10)	0.12	45.9% (44.6%, 47.1%)	6 (3, 10)	0.20
	Yes	555 (7.8%)	64.9% (60.7%, 68.8%)	6 (3, 11)		43.1% (38.9%, 47.3%)	6 (3, 11)	
No chronic health conditions	No^*^	4,701 (65.6%)	65.7% (64.3%, 67.0%)	6 (3, 10)	< 0.001	43.5% (42.1%, 44.9%)	6 (3, 10)	< 0.001
	Yes	2,460 (34.4%)	72.0% (70.2%, 73.8%)	6 (3, 10)		49.8% (47.8%, 51.8%)	6 (3, 10)	
Chronic health problems unknown	No^*^	6,032 (84.2%)	67.6% (66.4%, 68.8%)	6 (3, 10)	0.37	45.7% (44.5%, 47.0%)	6 (3, 10)	0.69
	Yes	1,129 (15.8%)	69.0% (66.2%, 71.7%)	6 (3, 10.5)		45.1% (42.2%, 48.0%)	6 (3, 10.5)	

^*^Reference category for each variable. p-values are from chi-square or Kruskal-Wallis tests comparing groups within each feature. Abbreviations: CI, confidence interval; GAD-7, Generalized Anxiety Disorder 7-item scale; HMO, Health Maintenance Organization; IQR, interquartile range; PHQ-9, Patient Health Questionnaire 9-item scale.

#### 2.6.1 Primary analysis

The association between baseline covariates and the rate of initial outcome (depression response or depression remission, modeled separately) was estimated using a log-linear model via Poisson regression (Laird and Olivier, 1981). Statistical testing revealed that the data did not satisfy the proportional hazards assumption required for Cox regression models, thus requiring a different modeling approach. The Poisson regression methodology provides an advantage over traditional Cox methods in that it is not necessary to assume proportional hazards, it creates the opportunity to directly estimate the underlying event rate, and allows the calculation of time-varying probabilities of the outcome rather than just hazard ratios on an unknown base rate (Efron, 1988). The rate ratios produced by Poisson models are also more intuitively interpretable than the odds ratios produced by logistic-based discrete time models. Follow-up time for the time-to-event models was defined as either the session number at which the outcome was observed or the session number from the last available PHQ-9 assessment within the observation window. Time-to-event data were structured in counting process notation using the following intervals: sessions 1–12, 13–24, 25–36, and 37–48; these represent the intervals in which a patient was actively in care and had potential to experience the outcome. Twelve session intervals were chosen to reasonably approximate phases of psychotherapy. Most symptom change in therapy happens within the first 8–16 sessions, with minimal change occurring after about 24–26 sessions (Robinson et al., 2020). At least one study used 12 sessions to demarcate two phases of therapy, with phase one occurring from sessions 1–12 and phase 2 from sessions 13–24 (Knipschild et al., 2023). Taken together, this literature suggests that 12 sessions represents meaningful incremental doses of psychotherapy.

As in traditional survival analysis, patients contributed only to session intervals in which they were present, and their time of removal from the population was accounted for in the duration of person-time contributed either by censoring or experiencing the incident outcome (Singer and Willett, 1991). The model allows calculation of the probability of achieving an outcome in each interval. Visualizations show how the probability of achieving response or remission changes as time in care increases.

#### 2.6.2 Case complexity feature selection

To avoid including unnecessary features and balance trade-offs between characterization and parsimony, we employed an elastic net procedure designed to retain model features which consistently appeared as effective predictors across many cross-validated replications. Consistent with the methodological literature on elastic net procedures, features were retained overall if they were retained in > 80% of replicates (Meinshausen and Buehlmann, 2009). In order to maintain factor integrity, if any level of a categorical variable was retained, the entire variable was retained. To enable comparison between the two outcomes (response and remission), any feature which was consistently identified as meaningful by the elastic net procedure for either response or remission was included in both models. The final model results were generated with unpenalized Poisson models. Any variables containing missing data used an explicit indicator variable for missingness, and the modal level of the variable was used as the reference category.

To illustrate how cumulative case complexity may influence time to outcome, three example patient profiles were created, representing the following three types of patients: the modal patient profile using the most frequently occurring characteristics, a more severe/complex profile, and a less complex profile. Factors varied in the profiles were identified as significant in the feature selection process and were validated by leveraging expert clinical opinion (authors NRF and MA). Probabilities of response and remission were calculated per interval for each of these hypothetical profiles.

#### 2.6.3 Secondary analysis

A secondary analysis evaluated whether patterns of earlier treatment response predict later treatment response for individuals who had not achieved clinical response or remission before session 24. To address this, we filtered the patients to those who had not achieved response (and separately remission) by 24 sessions and examined the binary outcome of whether the patient ever achieved the outcome within the observation window from sessions 25–48. In addition to baseline variables, we also included measures of clinical change prior to session 25: severity band of last known PHQ-9 prior to session 25, percent change from baseline score (represented in 10% change increments), and week-over-week variability in score change during sessions 1 through 24 (*z*-score scaled). The selection of 24 sessions was informed by the findings of Robinson et al. (2020) and clinical practice at the study site.

The secondary analysis was performed using the previously described elastic net methodology for feature selection and a log-linked Poisson model with a binary outcome, which provides coefficients that, once exponentiated, represent prevalence ratios for the prevalence of achieving the outcome within this window, given survival to session 24. All analyses were conducted using R version 4.4.3 (R Core Team, 2025).

## 3 Results

**Figure 1:**
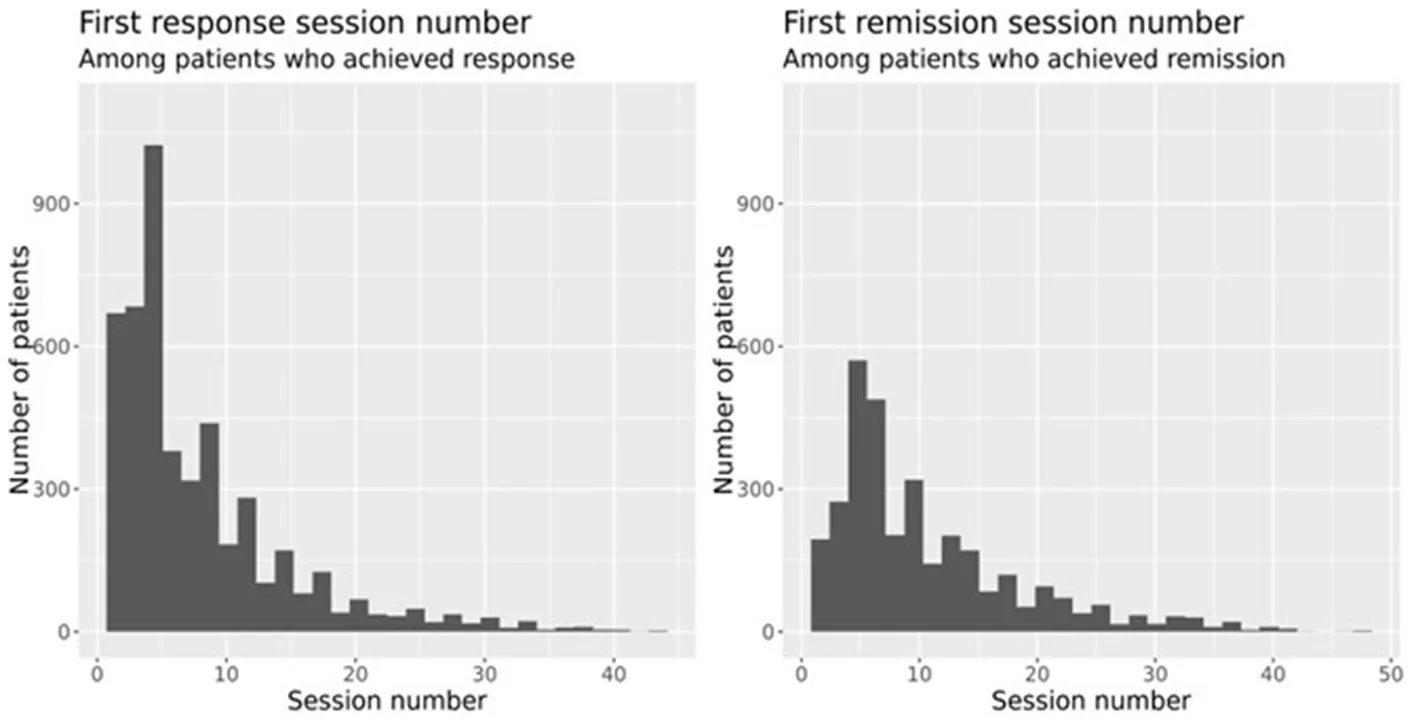
Distribution of initial response and remission by session number among patients who experienced these outcomes.

Among the 7,161 patients in the analysis, 67.9% achieved response and 45.6% achieved remission at least once during the period of observation, occurring on average at session 8.0 for response and session 10.6 for remission. The median outcome response and remission attainment was sessions 6 and 8, respectively. Figure 1 shows the distribution of initial occurrence of response and remission across sessions. Most initial occurrences occurred prior to session 12 (81.9% for response, 70.2% for remission) and the vast majority occurred by session 24 (96.0% for response, 92.6% for remission). Among patients who achieved initial response, 81% were still present in care at least four sessions later and 53% maintained response. Among patients who achieved remission, 74% were present at least four sessions later and 45% maintained response. Among these same patients who achieved an initial outcome, 66% of initial responders were in a state of response at their final session within the observation window and 59% of patients with initial remission were in a state of remission at their final session.

Table 1 describes baseline characteristics of patients in the analysis, who were diverse in complexity, severity, and across other self-reported sociodemographic characteristics. Many of these baseline features have significant univariate relationships with the presence of either response or remission, but the magnitude of these individual effects is small. Most effects only vary in frequency of response/remission by < 5–10 percentage points and in most cases, median time to outcome differs by approximately one session. A few notable exceptions are race/ethnicity, employment status, health status, baseline GAD, and chronic pain, each of which showed somewhat stronger univariate associations with time to outcome. The effect of these factors on response and remission at the last available session are listed in Supplementary Table 1.

### 3.1 Primary analysis

**Figure 2:**
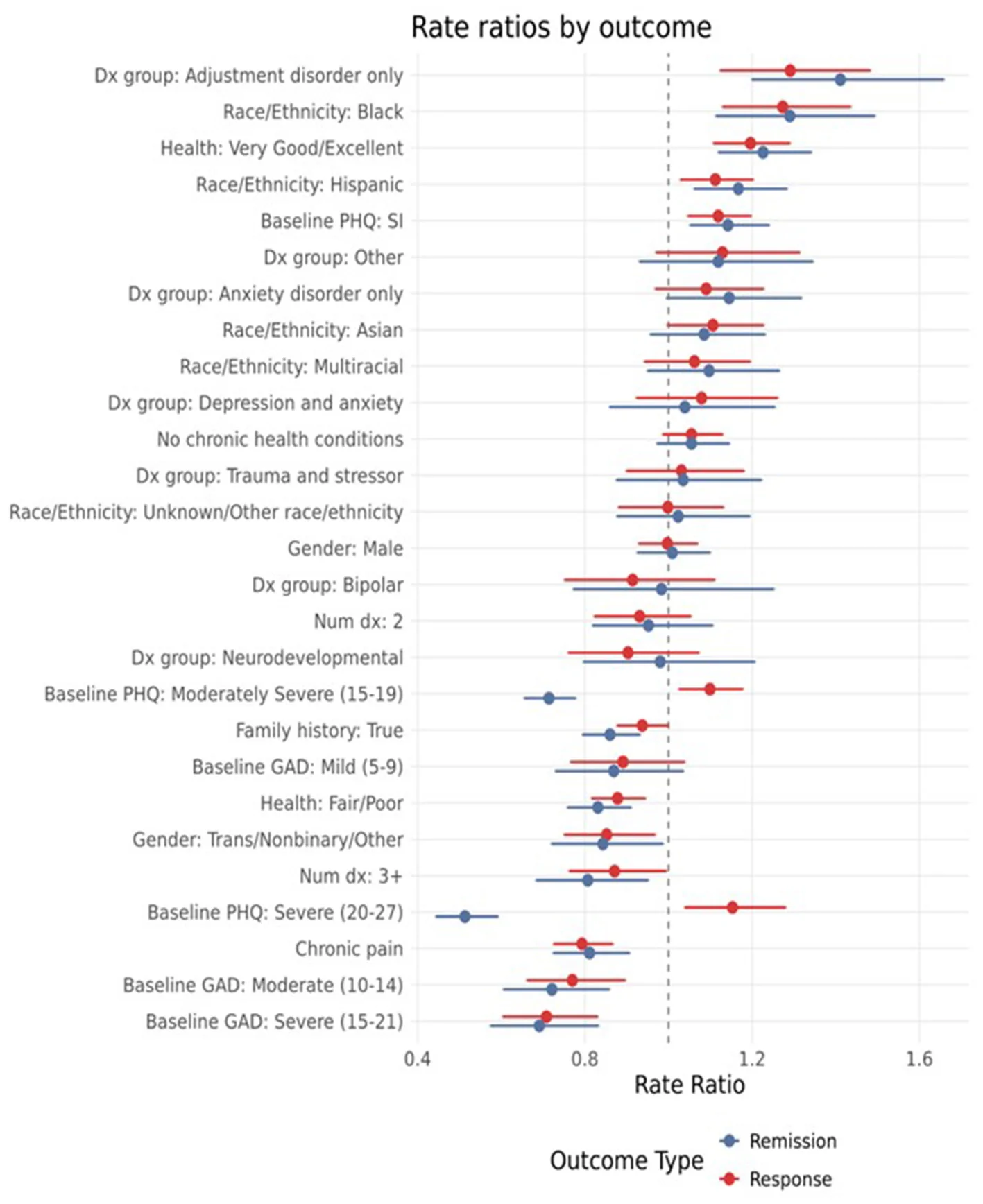
Rate ratios and 95% confidence intervals for the association between baseline patient factors and the rate (per unit clinical time) of incident initial response and remission.

The replicated elastic net procedure resulted in the following retained model factors: baseline PHQ-9, baseline GAD-7, presence of suicidal ideation (SI), diagnosis group, number of diagnoses, gender, race/ethnicity, self-reported health category, family history of mental illness, self-reported chronic pain, and self-reported absence of chronic health conditions. Rate ratios and 95% confidence intervals describing how baseline factors affect the rate (per unit clinical time) of response and remission are shown in Figure 2. The features most strongly associated with rate of response and remission were baseline severity of depressive symptoms, baseline SI, baseline severity of anxiety symptoms, self-reported health, self-reported chronic pain, number of diagnoses, family history, and some levels of diagnosis group, race, and ethnicity. In most cases, the association between the patient feature and the outcome was similar between response and remission, with one notable difference: higher baseline PHQ-9 was strongly positively associated with response and strongly negatively associated with remission.

**Figure 3:**
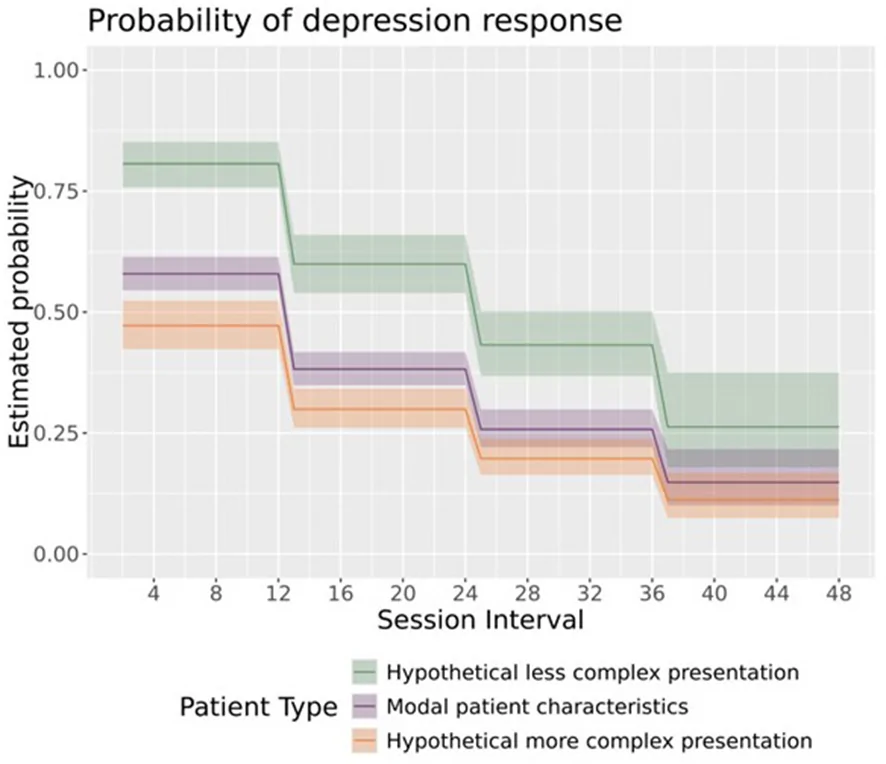
Estimated probability of depression response by session interval with confidence intervals for three hypothetical patient complexity profiles. The probability within each session interval represents the probability of the outcomes for all uncensored individuals in the interval.

**Figure 4:**
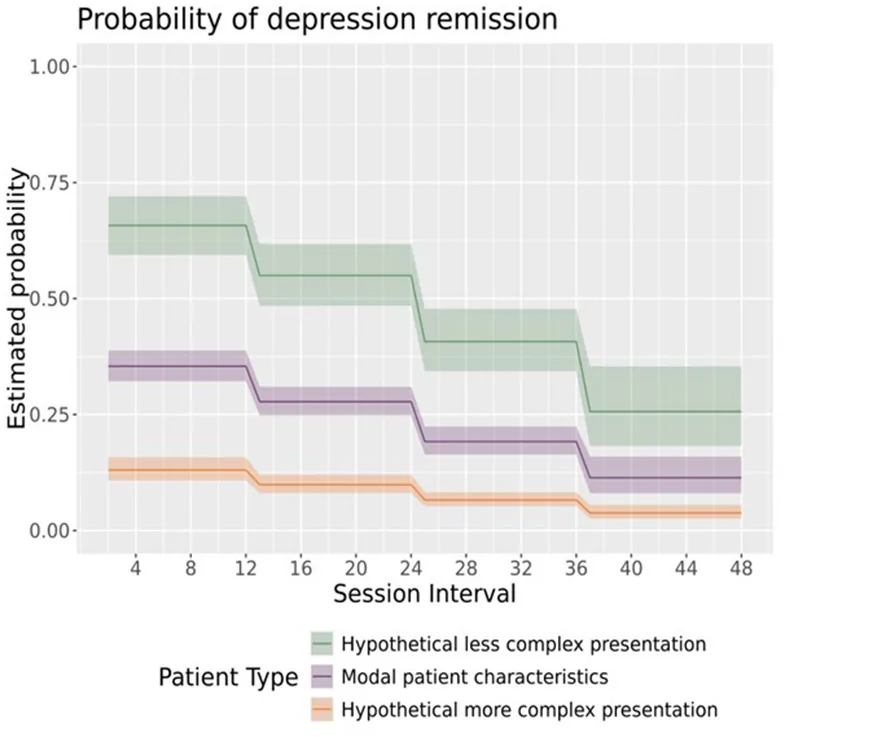
Estimated probability of depression remission by session interval confidence intervals for three hypothetical patient complexity profiles. The probability within each session interval represents the probability of the outcomes for all uncensored individuals in the interval.

To better characterize how these various features combine, three hypothetical patient profiles were constructed to predict likely time to outcomes for complex, modal, and less complex patient types (Table 2). Model predictions based on these profiles were used to calculate the probability of response and remission (separately) in each of the session intervals (Figures 3, 4). These figures demonstrate substantial variation in the probability of positive response in the first 12 sessions. In the first 12 sessions, the most complex patient profile had a predicted probability of only 47% response, modal patients a predicted probability of 57%, and less complex patients a predicted probability of 81%. Figure 4 demonstrates the large gap in probability of remission between patients of different complexity profiles, with complex patients having a lower likelihood of experiencing remission at any interval.

**Table 2:** Hypothetical patient profiles.

Patient feature	Less complex presentation	Modal presentation	More complex presentation
Baseline PHQ-9 range	Moderate (10–14)	Moderate (10–14)	Severe (20–27)
Baseline GAD-7 range	Mild (5–9)	Moderate (10–14)	Severe (15–21)
Baseline suicidal ideation	No	No	Yes
Diagnosis group	Adjustment only	Depression and anxiety	Trauma and stressor disorder
Number of diagnoses	1	2	3+
Overall health	Very good/excellent	Good	Fair/poor
Chronic pain	No	No	Yes
Relationship status	Partnered/married	Partnered/married	Single
Family history	No	Yes	Yes

Held fixed at reference/modal levels: Race/ethnicity = White, Gender = Woman, No chronic health conditions = false.

### 3.2 Secondary analysis

**Figure 5:**
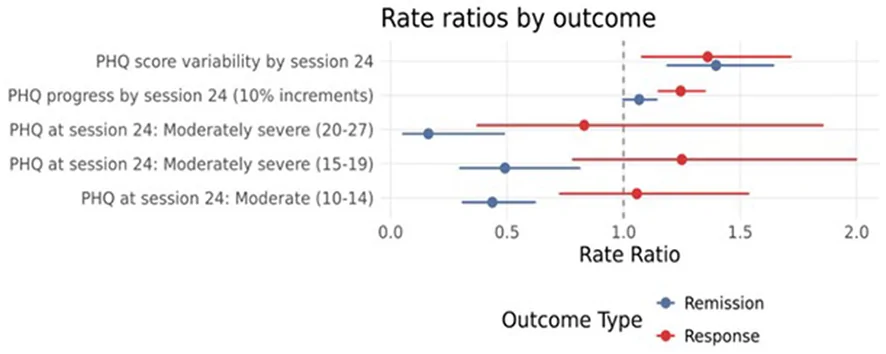
Rate ratios and 95% confidence intervals describing the association between patient features and rate of outcome (per unit clinical time) for individuals who did not achieve response or remission before session 24.

The secondary analysis aimed to address whether it is possible to predict which patients who have not achieved response or remission before session 25 may achieve outcomes in the remaining follow-up period. Of the original 7,161 patients, *n* = 778 (7.7%) had not yet achieved response but remained in care, and *n* = 1,477 (12.0%) had not yet achieved remission but remained in care. The replicated elastic net procedure used for feature selection yielded only features related to PHQ-9 change: symptom status prior to session 25, symptom reduction since baseline, and week-over-week symptom variability since baseline. No baseline patient features were retained in the feature-reduced set (Figure 5).

Higher week-over-week PHQ-9 score variability was associated with an increased likelihood of achieving both response and remission, as was each additional 10% gain in PHQ-9 symptom progress prior to session 25. Higher levels of PHQ-9 severity prior to session 25 were associated with reduced likelihood of remission in subsequent sessions.

## 4 Discussion

The aim of the study was to identify factors that could support clinicians, patients, and health systems in making treatment decisions for individuals undergoing psychotherapy for depression. This study used a large (*n* = 7,161) naturalistic sample of patients receiving psychotherapy for depression to evaluate the relationship between patient characteristics and time to clinical response and remission, and further, to evaluate whether patterns of change in symptoms can help supplement outcome prediction after 24 sessions of care. The results suggest that both case complexity and patterns of symptom change may be helpful in estimating the duration of treatment needed for patients to reach a positive clinical outcome; however, the value of this information varies depending on when it is being used in the course of care.

The study's findings are generally consistent with other literature on symptom change in depression. On average, patients showed a high probability of response early and then a decreasing probability of response as time in treatment increased. On aggregate, 81.9% of responders achieved response by session 12 and 96% by session 24. Treatment was also effective in general, supporting the finding that psychotherapy—particularly that supported by a robust measurement-based care implementation—is an effective treatment for depression (Forand et al., 2025a,b).

Also consistent with previous studies, we found that individual patient characteristics predicted significant variation in the probability of response and remission over time. Positive predictors included adjustment disorder, self-reported good or excellent health, identifying as Black or Hispanic/Latino, and the presence of suicidal ideation. The presence of suicidal ideation as a positive predictor of response and remission is somewhat surprising, but may be attributable to regression to the mean, as patients are likely to seek out treatment at their most distressed. Past research has shown that depressed patients with SI may attain nearly as good treatment outcomes as non-suicidal patients (von Brachel et al., 2019) and often experience reductions in both suicidal and depressive symptoms through treatment (Schneider et al., 2020).

Predictors of a longer time to response and remission included the presence of comorbid anxiety, presence of chronic pain, poor self-rated health, and diagnostic complexity (i.e., having three or more mental health diagnoses). Our findings are consistent with previous studies on chronic pain (Williams et al., 2021), comorbid anxiety (Coplan et al., 2015), poor self-rated health (Sato et al., 2013), and diagnostic complexity (Rush et al., 2022). These findings are in line with the case complexity hypothesis, in that the presence of more symptoms, conditions, or distress reduces the chance of a positive treatment outcome.

Overall, the impact of any individual baseline patient characteristic on depression response or remission was relatively small; however when the cumulative effect of prognostic characteristics were modeled, the results were striking. The lower complexity profile had a response probability of 81% over the first 12 sessions compared to the higher complexity profile, which had only a 47% probability.

This result supports the case complexity hypothesis and suggests that estimates of duration of care must be based on the holistic clinical picture rather than individual factors.

The impact of case complexity was most pronounced early in care. After 24 sessions in care, no baseline characteristics remained predictive of subsequent response or remission. Instead, only the magnitude of change in depression from baseline, current depression severity, and variability in depression symptoms from sessions 1–24 were significant predictors of response and remission beyond session 24. This set of findings suggests that it is the individuals who have shown some evidence of responsiveness to treatment (sometimes called “pliability”; DeRubeis et al., 2014) who are more likely to respond after a prolonged period of non-response. This is consistent with literature suggesting that individuals with chronic or long-standing depressive illness, or those who show minimal variability in their symptom presentation, may be particularly treatment resistant (Vittengl et al., 2016; Schumacher et al., 2024; Kube et al., 2025).

### 4.1 Implications for clinical decision-making

The findings in this paper suggest that different kinds of information about prognosis may be important at different points in care. Information about complexity, collected prior to treatment, may be most useful in selecting the modality or intensity of initial treatment strategies—for example, between lower or higher intensity psychotherapy (Delgadillo et al., 2022), or between psychotherapy alone or psychotherapy combined with medications. This strategy may optimize the chances for a positive outcome for those most in need while minimizing harms and costs for individuals likely to respond to a lower intensity treatment strategy (Forand et al., 2013). Further, clinicians may attend to specific risk factors that may interfere with treatment progress in depression, for example selecting treatment strategies for managing comorbid chronic pain, health concerns, or concurrent anxiety, or ensuring that these conditions are properly treated by other collaborating professionals. Additionally, the more complexity factors present within any individual, the greater the importance of setting appropriate expectations for treatment and engaging in collaborative decision-making with the patient around whether more aggressive or intensive treatment options would be preferred at the outset.

As treatment progresses, complexity factors may become less important for clinical decision-making. Our findings suggest that psychotherapy for depression may reach a point of diminishing returns by session 24 for most individuals. At this point in care, clinicians are advised to re-evaluate the treatment plan to determine whether continued care using the same approach or modality is warranted. In particular, individuals with a stagnant or stable and high pattern of depression symptoms week after week are strong candidates for a switch to a different or more intensive course of care (e.g., neuromodular treatments, intensive outpatient programs, etc.). In contrast, those individuals who have demonstrated some evidence of symptom improvement or variability over the first 24 sessions may still benefit from continuing the same course of treatment. This finding also highlights the importance of gathering data on the course of individual response to treatment to enable decisions as care progresses; these patterns can only be observed with continuous feedback on patient response to care.

Gathering feedback on treatment response over time and adjusting the course of care based on these insights is a core tenet of measurement-based care (MBC; Schuckard et al., 2017). The MBC literature suggests that when clinicians are responsive to cases that are “off-track”—meaning either those who are worsening or not changing as expected—patients have better outcomes (Shimokawa et al., 2010). Providers who use measurement-based care may combine model-based “case complexity” insights with the ongoing information gathered during a course of care to refine treatment plans and set expectations with patients about their likely care trajectory. In doing so, the results of this study can strengthen patient/provider communication and support therapeutic alliance (Aafjes-van Doorn et al., 2024), which may improve patient outcomes. These insights may also support more rapid decision-making about changes in the treatment plan, treatment modality, or level of care for patients at risk of non-response, which may in turn improve outcomes and reduce the overall costs and burden of depression.

### 4.2 Implications for clinicians, health systems, and payers

The findings in this paper may have implications across a range of stakeholders. Clinicians, health systems, and payers may observe that the “optimal” dose of care for individuals with depression engaged in psychotherapy appears to be around 12–24 sessions, by which point approximately 96% of responders will have achieved this outcome at least once. These stakeholders may use this information to estimate the expected duration of psychotherapy for most individuals with depression. The findings may also provide information that may be helpful in identifying which individuals may receive benefit beyond 24 sessions, which may be used in decision-making about the necessary duration of care. These findings suggest that the effectiveness—and perhaps cost-effectiveness—of treatment for depression varies by individual patient characteristics, suggesting that simple models allocating treatment resources to populations (e.g., session limits) are likely inadequate to fully realize the benefit of psychotherapy for depression. Stakeholders attempting to understand and predict the expected duration of care in depression in naturalistic settings should account for initial case complexity and course of symptom change (when possible) to provide more personalized estimates.

### 4.3 Strengths of the current study

To our knowledge, this is the first study in a large, diverse, naturalistic sample to examine the impact of both case complexity and patterns of symptom change as complementary predictors of response and remission in depression. The providers included in the study are heterogeneous with respect to their clinical approach and choice of therapeutic modality. Further, the study's dataset included symptom measurements taken at every treatment session with a 95% completion rate, indicating a robust MBC infrastructure and adherence (Forand et al., 2025a,b). The robust nature of the sample, the diversity of the patients and clinicians, and the naturalistic setting suggest that these results may be generalizable and therefore of use to clinicians, health systems, and payers.

### 4.4 Limitations

The study has the following limitations. First, most intake data and all data collected on the course of depression in care were self-reported, raising concerns about reliability and validity. Further, the study only examined time to the initial evidence of response and remission and did not examine the maintenance of these outcomes. Additional work is needed to explore maintenance of response to fully understand the durability of the treatment effects. Similarly, the study did not evaluate whether additional time in care is required for patients to maintain their clinical response, and if so, whether patient characteristics are associated with the duration of this interval. In addition, the reliability of response and remission probability estimates for patients in care after 24 sessions may be limited by the substantially lower sample sizes during those time periods. Although generally the domains of complexity factors observed to influence treatment outcomes in this study are consistent with other studies, (e.g., demographic factors, severity, comorbidity; Delgadillo et al., 2017), the exact associations between complexity variables and outcomes vary across studies. The specific associations observed will likely vary based on the how these variables are measured, the population, the therapists, and the treatments used. Thus while the concept of “case complexity” may be robust and replicable, the specific variables and associations observed in this study are unlikely to generalize to other settings. Those wishing to use case complexity in clinical decision-making may choose to construct models that are specific to their own clinical settings. The setting may also further impact the generalizability of the findings; specifically, therapists in this setting are trained in measurement-based care and may have outcomes that are superior to standard care settings, and the sample tends to have lower rates of dropout than other naturalistic samples (Forand et al., 2025a,b). Finally, variability in therapist effectiveness and the quality of psychotherapy delivered may also have an impact on these estimates over and above that which is predicted solely by patient characteristics. Indeed, on average, therapist effects account for about 5% of variability in outcomes (Johns et al., 2019), indicating that future work should attempt to estimate both therapist and patient characteristic contributions to outcomes.

### 4.5 Conclusion

This study set out to integrate insights from prior research on case complexity and trajectory of change and use these insights to explore patterns of response and remission within a large, naturalistic treatment setting using data collected in the normal course of clinical care. We found that indicators of case complexity as well as information about treatment response over time independently predict the probability of response and remission at different times during treatment. This study adds to the growing body of literature on the heterogeneity of treatment response in depression and helps advance efforts to personalize and refine treatments for depression.

## Data Availability

The data analyzed in this study is subject to the following licenses/restrictions: The datasets analyzed for this study are not publicly available due to patient privacy restrictions. Requests to access the data should be directed to the corresponding author. Requests to access these datasets should be directed to Nicholas Forand nforand@twochairs.com.

## References

[B1] Aafjes-van Doorn K. Spina D. S. Horne S. J. Békés V. (2024). The association between quality of therapeutic alliance and treatment outcomes in teletherapy: a systematic review and meta-analysis. Clinic. Psychol. Rev. 110:102430. doi: 10.1016/j.cpr.2024.102430 38636207

[B2] American Psychological Association (2019). APA Clinical Practice Guideline for the Treatment of Depression Across Three Age Cohorts: (505892019–001). Washington, DC: American Psychological Association.

[B3] Anderson E. M. Lambert M. J. (2001). A survival analysis of clinically significant change in outpatient psychotherapy. J. Clin. Psychol. 57, 875–888. doi: 10.1002/jclp.1056.abs 11406801

[B4] Bosley H. G. Soyster P. D. Fisher A. J. (2019). Affect dynamics as predictors of symptom severity and treatment response in mood and anxiety disorders: evidence for specificity. J. Pers Oriented Res. 5, 101–113. doi: 10.17505/jpor.2019.09 33569146 PMC7842610

[B5] Buckman J. E. J. Saunders R. Cohen Z. D. Barnett P. Clarke K. Ambler G. . (2021). The contribution of depressive ‘disorder characteristics' to determinations of prognosis for adults with depression: an individual patient data meta-analysis. Psychol. Med. 51, 1068–1081. doi: 10.1017/S0033291721001367 33849685 PMC8188529

[B6] Coplan J. D. Aaronson C. J. Panthangi V. Kim Y. (2015). Treating comorbid anxiety and depression: psychosocial and pharmacological approaches. World J. Psychiatry 5, 366–378. doi: 10.5498/wjp.v5.i4.366 26740928 PMC4694550

[B7] Cuijpers P. (2017). Four decades of outcome research on psychotherapies for adult depression: an overview of a series of meta-analyses. Can. Psychol. / Psychol. Can. 58, 7–19. doi: 10.1037/cap0000096

[B8] Cuijpers P. Karyotaki E. Weitz E. Andersson G. Hollon S. D. van Straten A. (2014). The effects of psychotherapies for major depression in adults on remission, recovery and improvement: a meta-analysis. J. Affect. Disord. 159, 118–126. doi: 10.1016/j.jad.2014.02.026 24679399

[B9] Cuijpers P. Miguel C. Harrer M. Plessen C. Y. Ciharova M. Papola D. . (2023). Psychological treatment of depression: a systematic overview of a ‘meta-analytic research domain'. J. Affect. Disord. 335, 141–151. doi: 10.1016/j.jad.2023.05.011 37178828

[B10] de Beurs E. Bruinsma C. Warmerdam L. (2020). The relationship between clinical complexity, treatment dose and outcome in everyday clinical practice. Eur. J. Psychiatry 34, 90–98. doi: 10.1016/j.ejpsy.2019.12.005

[B11] Delgadillo J. Ali S. Fleck K. Agnew C. Southgate A. Parkhouse L. . (2022). Stratified care vs stepped care for depression: a cluster randomized clinical trial. JAMA Psychiatry 79, 101–108. doi: 10.1001/jamapsychiatry.2021.3539 34878526 PMC8655665

[B12] Delgadillo J. Huey D. Bennett H. McMillan D. . (2017). Case complexity as a guide for psychological treatment selection. Journal of Consulting and Clinical Psychology. J. Consult. Clin. Psychol. 85, 835–853. doi: 10.1037/ccp0000231 28857592

[B13] Efron B. (1988). Logistic regression, survival analysis, and the Kaplan-Meier curve. J. Am. Stat. Assoc. 83, 414–425. doi: 10.1080/01621459.1988.10478612

[B14] DeRubeis R. J. Cohen Z. D. Forand N. R. Fournier J. C. Gelfand L. A. Lorenzo-Luaces L. (2014). The personalized advantage index: Translating research on prediction into individualized treatment recommendations. A demonstration. PLoS One. 9:e83875. doi: 10.1371/journal.pone.0083875 24416178 PMC3885521

[B15] Forand N.R. DeRubeis R.J. Amsterdam J.D. (2013). “The Combination of Psychotherapy and Medication in the Treatment of Mental Disorders.” in Bergin and Garfield's Handbook of Psychotherapy and Behavior Change, 6th Edition (Hoboken, NJ: Wiley). p. 735–774.

[B16] Forand N. R. Nettiksimmons J. Anton M. Truxson R. Vanderwood K. Green B. (2025a). Depression and anxiety outcomes in a technology-enabled psychotherapy practice: retrospective cohort study. JMIR Format. Res. 9:e76264. doi: 10.2196/76264 41330576 PMC12709161

[B17] Forand N. R. Nettiksimmons J. Brownell A. Anton M. T. Truxson R. Green B. . (2025b). The impact of measurement based care at scale: examining the effects of implementation on patient outcomes and provider behaviors. Front. Health Serv. 5:1659238. doi: 10.3389/frhs.2025.1659238 41394311 PMC12698511

[B18] Greenberg P. Chitnis A. Louie D. Suthoff E. Chen S. Y. Maitland J. . (2023). The economic burden of adults with major depressive disorder in the United States (2019). Adv. Ther. 40, 4460–4479. doi: 10.1007/s12325-023-02622-x 37518849 PMC10499687

[B19] Hansen N. B. Lambert M. J. (2003). An evaluation of the dose–response relationship in naturalistic treatment settings using survival analysis. Ment. Health Serv. Res. 5, 1–12. doi: 10.1023/A:1021751307358 12602642

[B20] Johns R. G. Barkham M. Kellett S. Saxon D. (2019). A systematic review of therapist effects: a critical narrative update and refinement to Baldwin and Imel's (2013) review. Clin. Psychol. Rev. 67, 78–93. doi: 10.1016/j.cpr.2018.08.004 30442478

[B21] Klein T. Breilmann J. Schneider C. Girlanda F. Fiedler I. Dawson S. . (2024). Dose-response relationship in cognitive behavioral therapy for depression: a nonlinear metaregression analysis. J. Consult. Clin. Psychol. 92, 296–309. doi: 10.1037/ccp0000879 38829329

[B22] Knipschild R. Klip H. van Leeuwaarden D. van Onna M. J. R. Lindauer R. J. L. Staal W. G. . (2023). Treatment of multiple traumatized adolescents by enhancing regulation skills and reducing trauma related symptoms: rationale, study design, and methods of randomized controlled trial (the Mars-study). BMC Psychiatry 23:644. doi: 10.1186/s12888-023-05073-4 37667200 PMC10478292

[B23] Kroenke K. Spitzer R. L. Williams J. B. (2001). The PHQ-9: validity of a brief depression severity measure. J. Gen. Intern. Med. 16, 606–613. doi: 10.1046/j.1525-1497.2001.016009606.x 11556941 PMC1495268

[B24] Kube T. Rapo E. Glombiewski J. A. Rief W. (2025). How people with major depression adjust their expectations of future life events in response to other patients' reports of the positive effects of psychotherapy. Behav. Res. Therapy 189:104736. doi: 10.1016/j.brat.2025.104736 40188647

[B25] Laird N. Olivier D. (1981). Covariance analysis of censored survival data using log-linear analysis techniques. J. Am. Stat. Assoc. 76, 231–240. doi: 10.1080/01621459.1981.10477634

[B26] Lee A. A. Sripada R. K. Hale A. C. Ganoczy D. Trivedi R. B. Arnow B. . (2021). Psychotherapy and depressive symptom trajectories among VA patients: comparing dose-effect and good-enough level models. J. Consult. Clinic. Psychol. 89, 379–392. doi: 10.1037/ccp0000645 34124925 PMC9383046

[B27] Lewis C. C. Simons A. D. Kim H. K. (2012). The role of early symptom trajectories and pretreatment variables in predicting treatment response to cognitive behavioral therapy. J. Consult. Clin. Psychol. 80, 525–534. doi: 10.1037/a0029131 22730951

[B28] Li F. Jörg F. Merkx M. J. M. Feenstra T. (2023). Early symptom change contributes to the outcome prediction of cognitive behavioral therapy for depression patients: a machine learning approach. J. Affect. Disord. 334, 352–357. doi: 10.1016/j.jad.2023.04.111 37149055

[B29] Lutz W. Stulz N. Köck K. (2009). Patterns of early change and their relationship to outcome and follow-up among patients with major depressive disorders. J. Affect. Disord. 118, 60–68. doi: 10.1016/j.jad.2009.01.019 19217669

[B30] Meinshausen N. Buehlmann P. (2009). Stability Selection. arXiv, arXiv:0809.2932. Available online at: https://arxiv.org/abs/0809.2932 (Accessed February 01, 2026).

[B31] Mills J. A. Suresh V. Chang L. Mayes T. Croarkin P. E. Trivedi M. H. . (2022). Socioeconomic predictors of treatment outcomes among adults with major depressive disorder. Psychiatr. Serv. 73, 965–969. doi: 10.1176/appi.ps.202100559 35354325 PMC9629028

[B32] Owen J. Adelson J. Budge S. Wampold B. Kopta M. Minami T. . (2015). Trajectories of change in psychotherapy. J. Clinic. Psychol. 71, 817–827. doi: 10.1002/jclp.22191 26235730

[B33] Perlman K. Benrimoh D. Israel S. Rollins C. Brown E. Tunteng J. F. . (2019). A systematic meta-review of predictors of antidepressant treatment outcome in major depressive disorder. J. Affect. Disord. 243, 503–515. doi: 10.1016/j.jad.2018.09.067 30286415

[B34] Popkov A. A. Barrett T. S. Shergill A. Donohue M. Anderson R. J. Karlin B. E. (2025). Association between depression symptom severity and total cost of care: findings from a large, 2-year, claims-based, retrospective population health study. J. Affect. Disord. 368, 41–47. doi: 10.1016/j.jad.2024.09.056 39271070

[B35] R Core Team (2025). R: A Language and Environment for Statistical Computing. Vienna: R Foundation for Statistical Computing. Available online at: https://www.R-project.org/ (Accessed February 01, 2026).

[B36] Riera-Serra P. Navarra-Ventura G. Castro A. Gili M. Salazar-Cedillo A. Ricci-Cabello I. . (2024). Clinical predictors of suicidal ideation, suicide attempts and suicide death in depressive disorder: a systematic review and meta-analysis. Eur. Arch. Psychiatry Clin. Neurosci. 274, 1543–1563. doi: 10.1007/s00406-023-01716-5 38015265 PMC11422269

[B37] Robinson L. Delgadillo J. Kellett S. (2020). The dose-response effect in routinely delivered psychological therapies: a systematic review. Psychother. Res. 30, 79–96. doi: 10.1080/10503307.2019.1566676 30661486

[B38] Rush A. J. Sackeim H. A. Conway C. R. Bunker M. T. Hollon S. D. Demyttenaere K. . (2022). Clinical research challenges posed by difficult-to-treat depression. Psychol. Med. 52, 419–432. doi: 10.1017/S0033291721004943 34991768 PMC8883824

[B39] Sato S. Ishikawa S. I. Togasaki Y. Ogata A. Sato Y. (2013). Long-term effects of a universal prevention program for depression in children: a 3-year follow-up study. Child Adolesc. Ment. Health 18, 103–108. doi: 10.1111/j.1475-3588.2012.00665.x 32847290

[B40] Saunders R. Buckman J. E. J. Cape J. Fearon P. Leibowitz J. Pilling S. (2019). Trajectories of depression and anxiety symptom change during psychological therapy. J. Affect. Disord. 249, 327–335. doi: 10.1016/j.jad.2019.02.043 30802698 PMC6428692

[B41] Schneider R. A. Chen S. Y. Lungu A. Grasso J. R. (2020). Treating suicidal ideation in the context of depression. BMC *Psychiatry* 20:497. doi: 10.1186/s12888-020-02894-5 33032569 PMC7545544

[B42] Schuckard E. Miller S. D. Hubble M. A. (2017). Feedback-Informed Treatment: Historical and Empirical Foundations, Feedback-Informed Treatment in Clinical Practice: Reaching for Excellence. Washington, DC: American Psychological Association, 13–35. doi: 10.1037/0000039-002

[B43] Schumacher L. Klein J. P. Hautzinger M. Härter M. Schramm E. Kriston L. (2024). Predicting the outcome of psychotherapy for chronic depression by person-specific symptom networks. World Psychiatry 23, 411–420. doi: 10.1002/wps.21241 39279420 PMC11403179

[B44] Shimokawa K. Lambert M. J. Smart D. W. (2010). Enhancing treatment outcome of patients at risk of treatment failure: meta-analytic and mega-analytic review of a psychotherapy quality assurance system. J. Consult. Clinic. Psychol. 78, 298–311. doi: 10.1037/a0019247 20515206

[B45] Simon G. E. Khandker R. K. Ichikawa L. Operskalski B. H. (2006). Recovery from depression predicts lower health services costs. J. Clin. Psychiatry 67, 1226–1231. doi: 10.4088/JCP.v67n0808 16965200

[B46] Singer J. D. Willett J. B. (1991). Modeling the days of our lives: using survival analysis when designing and analyzing longitudinal studies of duration and the timing of events. Psychol. Bull. 110, 268–290. doi: 10.1037//0033-2909.110.2.268

[B47] Spitzer R. L. Kroenke K. Williams J. B. Löwe B. (2006). A brief measure for assessing generalized anxiety disorder: the GAD-7. Arch. Intern. Med. 166, 1092–1097. doi: 10.1001/archinte.166.10.1092 16717171

[B48] Stewart R. E. Chambless D. L. (2008). Treatment failures in private practice: how do psychologists proceed?. Prof. Psychol. 39, 176–181. doi: 10.1037/0735-7028.39.2.176

[B49] Stiles W. B. Barkham M. Wheeler S. (2015). Duration of psychological therapy: relation to recovery and improvement rates in UK routine practice. Br. J. Psychiatry 207, 115–122. doi: 10.1192/bjp.bp.114.145565 25953889

[B50] Stulz N. Lutz W. (2007). Multidimensional patterns of change in outpatient psychotherapy: the phase model revisited. J. Clin. Psychol. 63, 817–833. doi: 10.1002/jclp.20397 17674397

[B51] Stulz N. Lutz W. Kopta S. M. Minami T. Saunders S. M. (2013). Dose-effect relationship in routine outpatient psychotherapy: does treatment duration matter?. J. Couns. Psychol. 60, 593–600. doi: 10.1037/a0033589 23815633

[B52] Vittengl J. R. Clark L. A. Thase M. E. Jarrett R. B. (2016). Longitudinal social-interpersonal functioning among higher-risk responders to acute-phase cognitive therapy for recurrent major depressive disorder. J. Affect. Disord. 199, 148–156. doi: 10.1016/j.jad.2016.04.017 27104803 PMC4862892

[B53] von Brachel R. Teismann T. Feider L. Margraf J. (2019). Suicide ideation as a predictor of treatment outcomes in cognitive-behavioral therapy for unipolar mood disorders. Int. J. Clinic. Health Psychol. 19, 80–84. doi: 10.1016/j.ijchp.2018.09.002 30619501 PMC6300714

[B54] Williams Z. J. Everaert J. Gotham K. O. (2021). Measuring depression in autistic adults: psychometric validation of the beck depression inventory–II. Assessment 28, 858–876. doi: 10.1177/1073191120952889 32864993 PMC8522206

[B55] World Health Organization (2023). Depression Key Facts. Geneva. Available online at: https://www.who.int/news-room/fact-sheets/detail/depression (accessed February 20, 2026).

