# Supplementary material for: Patient and course of care factors impacting time to response and remission in psychotherapy for depression
Source: Front Psychol. 2026 Aug 11;17:1816893. doi: 10.3389/fpsyg.2026.1816893 (PMC13503364; doi:10.3389/fpsyg.2026.1816893)
Supplement: Supplementary file 1 [file Table_1.DOCX]

**Supplemental Table 1: Prevalence of response and remission at final session within observation window (up to and including session 48 within 1 year of follow-up).**

| **Feature** | **Level** | **Frequency**  **N (%)** | **Final session response**  **% (95% CI)** | **Final session response**  **p-value** | **Final session remission**  **% (95% CI)** | **Final session remission**  **p-value** |
| --- | --- | --- | --- | --- | --- | --- |
| Age | 18-29 years | 2288 (32.0%) | 45.1% (43.0%, 47.1%) |  | 26.1% (24.4%, 28.0%) | 0.61 |
|  | 30-49 years | 3386 (47.3%) | 45.3% (43.6%, 47.0%) | 0.95 | 27.4% (25.9%, 28.9%) |  |
|  | 50-69 years | 1274 (17.8%) | 44.6% (41.8%, 47.4%) |  | 28.0% (25.6%, 30.6%) |  |
|  | 70+ years | 213 (3.0%) | 46.5% (39.7%, 53.4%) |  | 27.7% (21.9%, 34.3%) |  |
| Gender | Female | 4291 (59.9%) | 45.3% (43.8%, 46.8%) | 0.001 | 26.6% (25.3%, 27.9%) | 0.01 |
|  | Male | 1621 (22.6%) | 47.9% (45.5%, 50.4%) |  | 29.7% (27.5%, 32.0%) |  |
|  | Trans/Nonbinary/Other | 418 (5.8%) | 37.6% (32.9%, 42.4%) |  | 22.2% (18.4%, 26.6%) |  |
|  | Unknown | 831 (11.6%) | 42.5% (39.1%, 45.9%) |  | 27.3% (24.3%, 30.5%) |  |
| Race/Ethnicity | Asian | 653 (9.1%) | 46.7% (42.8%, 50.6%) | 0.09 | 28.2% (24.8%, 31.8%) | 0.32 |
|  | Black | 463 (6.5%) | 47.9% (43.3%, 52.6%) |  | 28.9% (24.9%, 33.3%) |  |
|  | Hispanic | 1438 (20.1%) | 46.9% (44.3%, 49.5%) |  | 28.6% (26.3%, 31.0%) |  |
|  | Multiracial | 475 (6.6%) | 42.9% (38.5%, 47.5%) |  | 24.6% (20.9%, 28.8%) |  |
|  | White | 2926 (40.9%) | 44.5% (42.7%, 46.4%) |  | 26.3% (24.8%, 28.0%) |  |
|  | Other | 66 (0.9%) | 56.1% (43.3%, 68.1%) |  | 34.8% (23.8%, 47.7%) |  |
|  | Unknown race/ethnicity | 1140 (15.9%) | 42.7% (39.8%, 45.7%) |  | 26.5% (24.0%, 29.2%) |  |
| Profile Completion | Yes | 6375 (89.0%) | 45.3% (44.1%, 46.5%) | 0.41 | 27.0% (25.9%, 28.1%) | 0.51 |
|  | No | 786 (11.0%) | 43.8% (40.3%, 47.3%) |  | 28.1% (25.0%, 31.4%) |  |
| Insurance | HMO | 5339 (74.6%) | 46.3% (45.0%, 47.6%) | 0.001 | 28.0% (26.8%, 29.3%) | 0.001 |
|  | Commercial / Self-pay | 366 (5.1%) | 46.7% (41.5%, 52.0%) |  | 26.0% (21.6%, 30.8%) |  |
|  | Medicaid | 1017 (14.2%) | 39.6% (36.6%, 42.7%) |  | 22.3% (19.8%, 25.0%) |  |
|  | Medicare | 439 (6.1%) | 42.4% (37.7%, 47.2%) |  | 28.0% (23.9%, 32.5%) |  |
| Relationship status | Partnered/Married | 3431 (47.9%) | 46.6% (45.0%, 48.3%) | 0.002 | 28.3% (26.8%, 29.9%) | 0.00 |
|  | Separated/Divorced/Widowed | 549 (7.7%) | 49.5% (45.3%, 53.8%) |  | 30.4% (26.6%, 34.5%) |  |
|  | Single | 1943 (27.1%) | 42.7% (40.5%, 45.0%) |  | 23.9% (22.0%, 25.9%) |  |
|  | Unknown/Other | 1238 (17.3%) | 42.8% (40.0%, 45.6%) |  | 27.4% (24.9%, 30.0%) |  |
| Employment status | Full-time | 3620 (50.6%) | 46.8% (45.2%, 48.5%) | 0.07 | 27.9% (26.4%, 29.4%) | 0.35 |
|  | Caregiver | 93 (1.3%) | 49.5% (39.0%, 60.0%) |  | 21.5% (13.9%, 31.5%) |  |
|  | Disabled | 174 (2.4%) | 40.2% (33.0%, 47.9%) |  | 24.7% (18.6%, 31.9%) |  |
|  | Part-time | 678 (9.5%) | 42.5% (38.7%, 46.3%) |  | 25.2% (22.0%, 28.7%) |  |
|  | Retired | 336 (4.7%) | 46.7% (41.3%, 52.2%) |  | 30.1% (25.3%, 35.3%) |  |
|  | Student | 345 (4.8%) | 42.6% (37.4%, 48.0%) |  | 29.0% (24.3%, 34.1%) |  |
|  | Unemployed | 620 (8.7%) | 44.8% (40.9%, 48.9%) |  | 25.6% (22.3%, 29.3%) |  |
|  | Unknown | 1295 (18.1%) | 42.5% (39.8%, 45.3%) |  | 26.2% (23.8%, 28.7%) |  |
| Health status | Very Good/Excellent | 1423 (19.9%) | 53.0% (50.4%, 55.6%) |  | 35.5% (33.0%, 38.0%) | <0.001 |
|  | Good | 2764 (38.6%) | 46.0% (44.1%, 47.9%) | <0.001 | 28.0% (26.4%, 29.8%) |  |
|  | Fair/Poor | 2108 (29.4%) | 39.5% (37.4%, 41.6%) |  | 20.2% (18.5%, 22.0%) |  |
|  | Unknown | 866 (12.1%) | 43.3% (40.0%, 46.7%) |  | 27.4% (24.4%, 30.5%) |  |
| Family history of mental illness | No | 2180 (30.4%) | 49.9% (47.8%, 52.0%) | <0.001 | 31.6% (29.7%, 33.6%) | <0.001 |
|  | Yes | 4032 (56.3%) | 43.1% (41.5%, 44.6%) |  | 24.8% (23.5%, 26.2%) |  |
|  | Unknown | 949 (13.3%) | 42.9% (39.7%, 46.1%) |  | 26.6% (23.8%, 29.5%) |  |
| Baseline PHQ-9 | Moderate (10-14) | 4063 (56.7%) | 43.8% (42.3%, 45.4%) | 0.03 | 31.3% (29.9%, 32.7%) | <0.001 |
|  | Moderately Severe (15-19) | 2171 (30.3%) | 46.6% (44.5%, 48.7%) |  | 24.2% (22.4%, 26.1%) |  |
|  | Severe (20-27) | 927 (12.9%) | 47.5% (44.2%, 50.7%) |  | 15.7% (13.5%, 18.3%) |  |
| Baseline GAD-7 | Minimal (0-4) | 268 (3.7%) | 46.3% (40.2%, 52.4%) | 0.45 | 31.0% (25.6%, 36.9%) | <0.001 |
|  | Mild (5-9) | 1878 (26.2%) | 46.6% (44.3%, 48.9%) |  | 31.9% (29.9%, 34.1%) |  |
|  | Moderate (10-14) | 2776 (38.8%) | 44.8% (42.9%, 46.7%) |  | 27.6% (25.9%, 29.3%) |  |
|  | Severe (15-21) | 2239 (31.3%) | 44.2% (42.1%, 46.3%) |  | 22.1% (20.4%, 23.9%) |  |
| Baseline PHQ-9 | No suicidal ideation | 5226 (73.0%) | 44.2% (42.9%, 45.6%) | 0.01 | 27.5% (26.3%, 28.7%) | 0.26 |
|  | Any suicidal ideation | 1935 (27.0%) | 47.6% (45.4%, 49.9%) |  | 26.1% (24.2%, 28.2%) |  |
| Diagnosis group | Adjustment disorder only | 425 (5.9%) | 54.4% (49.5%, 59.1%) | <0.001 | 37.6% (33.1%, 42.5%) | <0.001 |
|  | Anxiety disorder only | 758 (10.6%) | 45.5% (41.9%, 49.1%) |  | 30.7% (27.5%, 34.2%) |  |
|  | Depression and anxiety | 2321 (32.4%) | 45.9% (43.8%, 47.9%) |  | 26.5% (24.8%, 28.4%) |  |
|  | Depression only | 931 (13.0%) | 45.5% (42.3%, 48.8%) |  | 27.1% (24.3%, 30.1%) |  |
|  | Bipolar | 269 (3.8%) | 42.0% (36.1%, 48.2%) |  | 21.2% (16.6%, 26.7%) |  |
|  | Neurodevelopmental | 473 (6.6%) | 38.1% (33.7%, 42.6%) |  | 24.1% (20.4%, 28.3%) |  |
|  | Trauma and stressor | 1258 (17.6%) | 43.1% (40.3%, 45.9%) |  | 24.7% (22.4%, 27.2%) |  |
|  | Other | 726 (10.1%) | 45.7% (42.1%, 49.4%) |  | 27.4% (24.2%, 30.8%) |  |
| Number of diagnoses | 1 | 2672 (37.3%) | 47.2% (45.2%, 49.1%) | 0.001 | 29.8% (28.1%, 31.6%) | <0.001 |
|  | 2 | 3582 (50.0%) | 44.7% (43.1%, 46.4%) |  | 26.4% (25.0%, 27.9%) |  |
|  | 3 | 806 (11.3%) | 41.3% (37.9%, 44.8%) |  | 22.3% (19.5%, 25.4%) |  |
|  | 4 | 96 (1.3%) | 35.4% (26.1%, 45.9%) |  | 16.7% (10.1%, 26.0%) |  |
|  | 5 | 5 (0.1%) | 60.0% (17.0%, 92.7%) |  | 60.0% (17.0%, 92.7%) |  |
| Diabetes | No | 6807 (95.1%) | 45.2% (44.0%, 46.4%) | 0.60 | 27.4% (26.3%, 28.4%) | 0.03 |
|  | Yes | 354 (4.9%) | 43.8% (38.6%, 49.1%) |  | 22.3% (18.2%, 27.1%) |  |
| Chronic pain | No | 5949 (83.1%) | 46.6% (45.4%, 47.9%) | <0.001 | 28.7% (27.5%, 29.8%) | <0.001 |
|  | Yes | 1212 (16.9%) | 37.7% (35.0%, 40.5%) |  | 19.5% (17.3%, 21.8%) |  |
| Heart Disease | No | 7061 (98.6%) | 45.3% (44.1%, 46.4%) | 0.03 | 27.3% (26.2%, 28.3%) | 0.002 |
|  | Yes | 100 (1.4%) | 35.0% (25.9%, 45.3%) |  | 16.0% (9.7%, 25.0%) |  |
| Cancer | No | 6963 (97.2%) | 45.2% (44.0%, 46.4%) | 0.63 | 27.0% (26.0%, 28.1%) | 0.32 |
|  | Yes | 198 (2.8%) | 43.4% (36.5%, 50.7%) |  | 30.3% (24.1%, 37.3%) |  |
| Respiratory Problems | No | 6606 (92.2%) | 45.4% (44.1%, 46.6%) | 0.20 | 27.2% (26.2%, 28.3%) | 0.51 |
|  | Yes | 555 (7.8%) | 42.5% (38.4%, 46.8%) |  | 25.9% (22.4%, 29.8%) |  |
| No chronic health conditions | No | 6032 (84.2%) | 45.3% (44.0%, 46.5%) | 0.62 | 27.3% (26.2%, 28.5%) | 0.37 |
|  | Yes | 1129 (15.8%) | 44.5% (41.5%, 47.4%) |  | 26.0% (23.5%, 28.7%) |  |
| Chronic health problems unknown | No | 4701 (65.6%) | 42.6% (41.1%, 44.0%) | <0.001 | 25.1% (23.9%, 26.3%) | <0.001 |
|  | Yes | 2460 (34.4%) | 50.0% (48.0%, 52.0%) |  | 31.0% (29.2%, 32.9%) |  |
